# Primary Effusion Lymphoma Cell Death Induced by Bortezomib and AG 490 Activates Dendritic Cells through CD91

**DOI:** 10.1371/journal.pone.0031732

**Published:** 2012-03-07

**Authors:** Mara Cirone, Livia Di Renzo, Lavinia Vittoria Lotti, Valeria Conte, Pankaj Trivedi, Roberta Santarelli, Roberta Gonnella, Luigi Frati, Alberto Faggioni

**Affiliations:** Department of Experimental Medicine, Istituto Pasteur-Fondazione Cenci Bolognetti, La Sapienza University, Rome, Italy; Institute of Cancerology Gustave Roussy, France

## Abstract

To understand how cytotoxic agent-induced cancer cell death affects the immune system is of fundamental importance to stimulate immune response to counteract the high mortality due to cancer. Here we compared the immunogenicity of Primary Effusion Lymphoma (PEL) cell death induced by anticancer drug Bortezomib (Velcade) and Tyrphostin AG 490, a Janus Activated Kinase 2/signal trasducer and activator of transcription-3 (JAK2/STAT3) inhibitor. We show that both treatments were able to induce PEL apoptosis with similar kinetics and promote dendritic cells (DC) maturation. The surface expression of molecules involved in immune activation, namely calreticulin (CRT), heat shock proteins (HSP) 90 and 70 increased in dying cells. This was correlated with DC activation. We found that PEL cell death induced by Bortezomib was more effective in inducing uptake by DC compared to AG 490 or combination of both drugs. However the DC activation induced by all treatments was completely inhibited when these cells were pretreated with a neutralizing antiboby directed against the HSP90/70 and CRT common receptor, CD91. The activation of DC by Bortezomib and AG 490 treated PEL cells, as seen in the present study, might have important implications for a combined chemo and immunotherapy in such patients.

## Introduction

Primary effusion lymphoma (PEL) is a non-Hodgkin's lymphoma characterized by lymphomatous effusions of pleural, pericardial and abdominal cavities [1]. It is characterized by a poor response to conventional chemotherapy and by an extremely aggressive clinical course. Its pathogenesis seems to be linked to an oncogenic virus, human herpesvirus 8 (HHV-8, also called KSHV, Kaposi's sarcoma associated herpes virus) [2]. Promising preliminary results in the PEL treatment have been obtained with Bortezomib, a proteasome inhibitor known to induce caspase-dependent apoptosis of PEL cells in vitro [3]. Bortezomib has received Food and Drug Administration (FDA) approval for the treatment of multiple myeloma [4], a disease that demonstrates some similarities with PEL, such as the constitutive activation of NF-Kappa B and STAT3. Tyrphostin AG 490, a JAK2/STAT3 inhibitor, has been also reported to induce caspase-dependent apoptosis in PEL [5]. STAT3 is indeed constitutively activated in this lymphoma and its growth seems to be dependent on the STAT3 signaling [5]. Although apoptosis has been for long time considered an apparently uniform and immunologically silent type of cell death, it is now evident that biochemical diversity exists that may render it immunogenic or not [6]. Diverse tumor cell type or the same tumor cell type dying in response to different cell death triggers can result in apoptosis that elicits a different activation of immune response [7]–[8].The immunizing properties of killed tumor cells depend on the ability of cytotoxic agents to render their death immunogenic so that the immune system can be specifically alerted to the presence of a tumor. The characteristics of the immunogenic cell death are the traslocation of the endoplasmic reticulum-resident CRT to the plasma membrane, followed by release or surface expression of HSP70 and HSP90 molecules that either provide a direct immunogenic signal for DC activation or act as vehicles for peptide antigen exposure [9]. The expression on the cell surface of several chaperones has been indicated as the most important mechanism for the activation of the immune system and in particular of the DC [10]. In particular, the cell surface exposure of CRT has been shown to be essential for the uptake of dying tumor cells by DC [11], while the exposure of HSP90 and HSP70 or their release promotes DC maturation [12]. CRT is traslocated on the plasma membrane following different types of endoplasmic reticulum (ER) stress stimuli including the ER stress determined by some chemotherapeutic agents [13]. HSP90 and HSP70 are two chaperone proteins that are also normally localized in the intracellular compartment where they play cytoprotective functions. Although the mechanisms underlying their membrane traslocation are not completely clear, it is known that their expression on the cell surface of stressed or dying cells has immunostimulatory properties towards immune cells such as Natural Killer (NK) and DC [14]. In this paper, we compared the proteasome inhibitor Bortezomib and the JAK2/STAT3 inhibitor tyrphostin AG 490 in triggering BC3 PEL apoptosis and subsequently in their ability to promote the DC maturation. Our results show that both drugs were able to induce BC3 apoptosis and DC maturation through traslocation of CRT and HSPs on the surface of dying cells. A previous study has highlighted the importance of chaperone traslocation also in vivo showing that, although displaying the same level of apoptosis or necrosis, indolent non-Hodkgin's lymphoma cells obtained from patients with a good response to chemotherapy were better able to translocate CRT and HSP90 to the cell surface than those of nonresponders [15]. We also compared the ability of the BC3 cells killed by these drugs to stimulate the uptake by DC and found that Bortezomib alone induced an higher percentage of phagocytosis. Beside the CRT and HSPs traslocation, the caspase activation usually present in the apoptotic process is important for the immunogenicity of the cell death [16].

The DC have a pivotal role in the eradication of apoptotic cancer cells. These cells are able to mediate the cross-presentation of tumor antigens to the cytotoxic T cells [17], so the DC activation by dying tumor cells is of fundamental importance for the outcome of cancer therapy [18]. Several receptors such as TLR4 and 2, CD14, Lox-1 have been reported to bind the chaperone molecules on the surface of DC [19]. Among them CD91 that has been reported by Basu et al. as the common receptor for HSP90, HSP70 and CRT [20]. In addition, it has been shown that viral and tumor antigens are cross-presented by DC through CD91pathway in patients with Kaposi sarcoma [21]. In the present study, we analyzed the effect of BC3 killed by Bortezomib and AG 490 in inducing DC activation. Furthermore, we evaluated if DC maturation is dependent on the exposure of CRT and HSPs, caspase activation and CD91 expression.

## Materials and Methods

### Ethics statement

The study was approved by the ethical Committee of Policlinico Umberto I, University of Rome “Sapienza”.

### Cells, reagents and antibodies

The PEL cell lines BC-3 and BCBL-1 (ATCC) were cultured in RPMI 1640 10% Fetal Calf Serum (FCS) (Euroclone), glutamine and antibiotics. Bortezomib (Velcade) was purchased from Millennium Pharmaceutical Inc. (Cambridge, MA, USA) and AG 490 was purchased from Calbiochem. Following monoclonal antibodies were used in this study: anti CD83 (Santa Cruz, Biotechnology incorporation), anti CD86 (BD Pharmingen), anti CD1a-PE (BD Pharmingen), anti Calreticulin (Thermo Scientific), anti HSP90 and anti HSP70 (Stressgen Biotech) and anti CD91 (Calbiochem).

### Generation of monocyte-derived DC

To generate monocyte-derived DC, human peripheral blood mononuclear cells, obtained from healthy donors were isolated by Fycoll-Paque gradient centrifugation (Pharmacia, Uppsala, Sweden) from buffy coats. CD14+ monocytes were positively selected using anti-CD14 MAb-conjugated magnetic microbeads (Miltenyi Biotec, Auburn, Calif.). Purified monocytes were cultured at a density of 1×10^6^ cells/3 ml in 12-well plates for 6 days in RPMI 1640 (Euroclone) containing 10% FCS, 2 mM L-glutamine, 100 U/ml penicillin G, 100 mg/ml streptomycin and recombinant human granulocyte-macrophage colony stimulating factor (GM-CSF) plus interleukin 4 (IL-4) (50 ng/ml and 20 ng/ml respectively, Miltenyi Biotec, Auburn, Calif.).

### Apoptosis assay

The effect of Bortezomib, Tyrphostin AG 490 or both was examined after 6, 12 and 24 hours of treatments. The cells were washed with ice-cold phosphate buffered saline (PBS), resuspended in annexin V binding buffer (BD Pharmingen, San Diego, CA), and subsequently stained with annexin V–fluorescein isothiocyanate (FITC) (BD Pharmingen) according to the manufacturer's recommendation. To assess the effect of the drugs on nuclear DNA fragmentation, hypodiploic (subG1) events were determined by cytofluorimetric analysis. Briefly, cells were washed with PBS and fixed in ice cold ethanol/water (70/30, v/v) for 1 hour, washed twice and resuspended in PBS, containing RNase, 40 ug/ml, and an equal volume of PBS containing propidium iodide (PI), 100 ug/ml. Events in the different cell cycle phases were gated manually. At least 10.000 events/sample were analysed using a cytofluorimeter EPICS XL Coulter (Hialeah, FL), equipped with the software “Multicycle for DNA content and cell cycle analysis” (Phoenix Flow System, San Diego, CA). The events in subG1, representing the apoptotic cells, are given as percentage of the total cell population. In the assays in which the caspase pathway was examined, the cell-permeable caspase inhibitor carbobenzoxy-valyl-alanyl-aspartyl (-methyl ester-fluoromethy ketone (Z-VAD-fmk) (Calbiochem) was added at 100 µM to the cell suspension before the induction of apoptosis.

### HSPs surface expression on dying tumor cells

PEL cells were treated by Bortezomib, AG 490 or both and the expression of surface CRT, HSP90 and HSP70 was analyzed 12 to 24 hours later by flow cytometry using specific monoclonal antibodies.

### Immunofluorescence

Untreated or treated PEL cells as described above, were fixed with 4% paraformaldehyde in PBS for 30 min, and to investigate CRT localization, the cells were labeled with the chicken anti human CRT antibody (Thermo Scientific). The primary antibody was visualized using Phycoerythrin (PE)- conjugated anti chicken antibody.

Cells were analyzed using an Apotome Axio Observer Z1 inverted microscope (Zeiss, Oberkochen, Germany), equipped with an AxioCam MRM Rev.3 at 40× magnification.

### Phagocytosis assay

The phagocytosis assay was performed as decribed previously [22]. Briefly, apoptosis was induced in 2×10^5^ CFMDA-labelled PEL cells by Bortezomib, AG 490 or both and, after washing, were co-cultivated with 2×10^5^ iDCs in 24 well plates for 4 hours in order to monitor phagocytosis. DC were labeled with anti-CD1a-PE antibody and phagocytosis of apoptotic cells was defined by the percentage of CFMDA-PE double positive cells by flow cytometric analysis.

### RNA interference (RNAi)

iDC were transfected with 50 pmols of CD91 siRNA or with 50 pmols of non-targeting siRNA (siCONTROL) (Santa-Cruz Biotechnology, Inc.) using Lipofectamine RNAiMAX transfection reagent (Invitrogen), according to the manufacture's instructions. The cells were incubated in a 5% CO_2_ incubator at 37° until they were assayed for gene knockdown 72 hours later. Prior to use in coculture with PEL the iDC were analyzed by Western blot and by FACS analysis for CD91 expression.

### FACS analysis of DC phenotype after interaction with killed tumor cells

Phenotype of DC cultured with tumor cells was monitored by flow cytometry. Tumor cells (BC3 and BCBL1) were treated with Bortezomib or AG 490 for 24 hours and then cocultured for 24 hours with immature DC (iDC). In some experiments, PEL cells were not washed to monitor the drug's side effect on DC. In neutralizing experiments, tumor cells were coated with anti-HSP90, anti-HSP70 and ani-CRT mAbs (10 µg/mL) or DC were incubated with anti azide-free CD91 (10 µg/mL) before setting up the coculture. To confirm the role of CD91 in some experiments the DC were teated with CD91 siRNA before the coculture with PEL. The phenotype of DC was monitored by staining with Phycoerytrin (PE)-conjugated anti CD83 and FITC-conjugated anti CD86 for 30 minutes at 4°C, washed twice in PBS and analyzed on a flow cytometer. DC were gated according to their FSC and SSC properties. Appropriate isotype controls were included and 5000 viable DC were acquired for each experiment.

### Immunoblot analysis

PEL cells treated by Bortezomib, AG 490 or both were lysed in a lysis buffer containing 50 mM Tris HCl, pH 6.8, 10% glycerol and 2% SDS. The lysates were then resolved by SDS-PAGE and transferred to nitrocellulose membranes (Bio-Rad). The levels of HSP90 and HSP70 were detected using specific antibodies. The DC untreated or treated with CD91siRNA or with non-targeting siRNA (siCONTROL) were lysed in the above lysis buffer, resolved by SDS-PAGE, transferred to nitrocellulose membrane and probed with anti-CD91 monoclonal antibody. β-actin was also verified as loading control. The reactions were visualized by enhanced chemiluminescence (Amersham).

## Results

### Bortezomib and AG 490 induce apoptosis in PEL with similar kinetic

**Figure 1:**
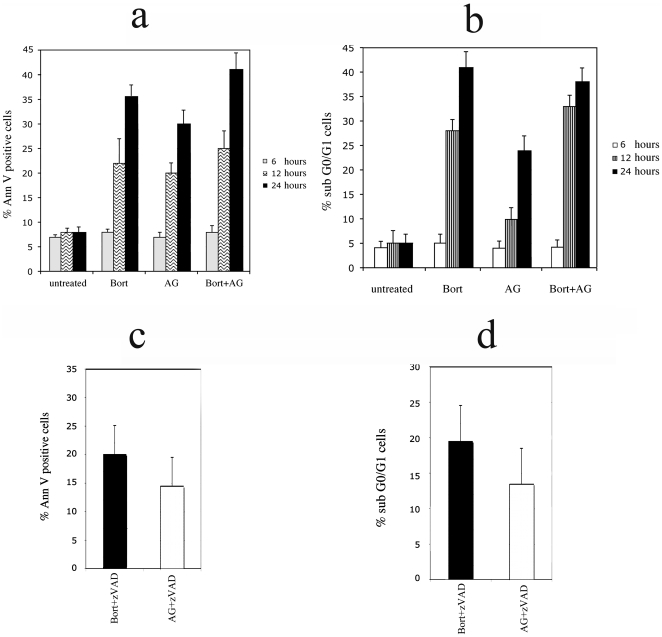
Bortezomib and AG 490 induce apoptosis in BC3 PEL cell line. PEL cells were exposed to Bortezomib, AG 490 or both for the indicated times, then resuspended in Annexin V binding buffer and stained with Annexin V (a). The effect of the drugs was also evaluated on nuclear fragmentation (sub-G_0_/G_1_ phase) that represent the apoptotic cells. Cells were fixed in ethanol/water (70/30, vol/vol), washed in PBS containing RNAse and then stained with Propidium Iodide (PI) (b). The analysis was performed by flow cytometry. The reduction of the percentage of Annexin V positive (c) or the sub-G_0_/G_1_ cells (d), obtained with Z-VAD-fmk pre-treatment at 100 µM, is also shown. The experiments were performed simultaneously with 1a and 1b at 24 hours. Mean plus SD is indicated.

We first compared the effect of Bortezomib and AG 490 in BC3 PEL apoptosis. The drug concentrations used were 25 nM for Bortezomib and 100 µM for AG 490 and were chosen according to previous studies [3], [5] and confirmed by pilot experiments (data not shown). BC3 were treated with Bortezomib and AG 490, alone or in combination and the induction of apoptosis was assessed by the percentage of Annexin V positive cells after 6, 12 and 24 hours treatment. As shown in figure 1a Bortezomib induced an higher percentage of Annexin V positive cells in comparison to AG 490 that increased with the combination of both drugs, suggesting a synergistic effect on BC3 apoptosis. Similar results were obtained with BCBL1 PEL cell line (data not shown). The induction of apoptosis was also confirmed by the appearance of an higher percentage of events with sub G0/G1 DNA content, as mesured by PI staining (figure 1b). Subsequently, BC3 pre-treatment with pancaspase inhibitor Z-VAD-fmk showed reduction of Bortezomib- or AG 490-induced apoptosis (figure 1c and d).

### Apoptotic PEL cell lines induced DC maturation and DC phagocytosis

**Figure 2:**
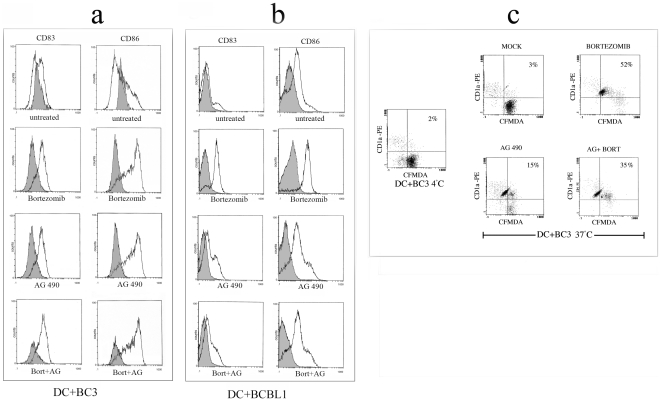
Effect of apoptotic BC3 and BCBL1 cells on DC. CD83 and CD86 expression was verified by FACS analysis as indicator of DC maturation induced with BC3 cells (a) and BCBL1 (b) treated with Bortezomib, AG 490 or both for 24 hours and cocultured with iDC for 12 hours. Staining with isotype control (filled histograms) is also shown. Phagocytosis of Bortezomib and AG 490 treated BC3 cells (stained with CFMDA-green) by DC (stained with anti-CD1a/PE-red) in a four hour assay. The phagocytosis of apoptotic cells is indicated by the percentage of CFMDA-PE double positive cells by flow cytometric analysis (c). Results are representative of three independent experiments.

Once assessed that both treatments were able to induce BC3 PEL apoptosis, we next tested the ability of live or apoptotic BC3 cells treated with Bortezomib and AG 490 to induce DC maturation after 12 hours of coculture with immature DC (iDC). Following this, the DC were stained with a cocktail of mAbs and analyzed by FACS analysis for the expression of CD83 maturation marker and CD86 costimulatory molecule. DC were gated according to their FSC and SSC properties. The exposure to BC3 cells induced to apoptosis by Bortezomib and to a lesser extent by AG 490 for 24 hours, resulted in a substantial up-regulation of CD86 and a marginal increase of CD83 compared to the exposure of iDC to the untreated BC3 cells (figure 2a). We also verified if the same activating effect on DC could be obtained by coculturing them with BCBL1 treated with Bortezomib, AG 490 or both. As shown in figure 2b, with this PEL cell line we obtained a comparable effect on DC activation, as indicated by CD86 and CD83 expression. However it is important to point out that the DC exposed to the untreated BC3 or BCBL1 cells did show a modest activation of DC. The combination of both drugs, although induced an higher percent of apoptotic cells (figure 1a and b) did not have synergistic effect on DC activation (figure 2a and b). Interestingly, we found that Bortezomib-treated BC3 cells exerted a toxic effect on DC that could be overcome by extensive washes prior to the cocultures. In contrast, AG 490 treatment did not have any toxic effect on DC (data not shown). We next evaluated whether the DC maturation induced by BC3 cell death was caspase-dependent. Z-VAD-fmk pre-treatment reduced drug-induced PEL apoptosis (see above) and slightly inhibited the activation of DC (data not shown). We finally tested the effect of Bortezomib- and AG 490-induced BC3 PEL apoptosis on DC phagocytic ability. To this end, dye CFMDA-labelled BC3 cells untreated or treated with Bortezomib and AG 490 alone or in combination were fed to immature DC (iDC). After 4 hours of incubation, the DC were stained with anti-CD1a mAb and analysed by FACS for the appearance of CFMDA-PE double-positive cells, indicating phagocytosed cells. The results show an increased phagocytosis by DC of BC3 cells killed by Bortezomib and to a lesser extent by AG 490 or combination of both in comparison to untreated BC3 cells (figure 2c). Altogether, these data indicate that both Bortezomib-and AG 490-induced PEL apoptosis triggered DC maturation, however AG 490, was less able in inducing DC uptake of apoptotic cells.

### Bortezomib and AG 490 treatments induced traslocation of CRT, HSP90 and HSP70 on the cell surface of dying PEL cells

**Figure 3:**
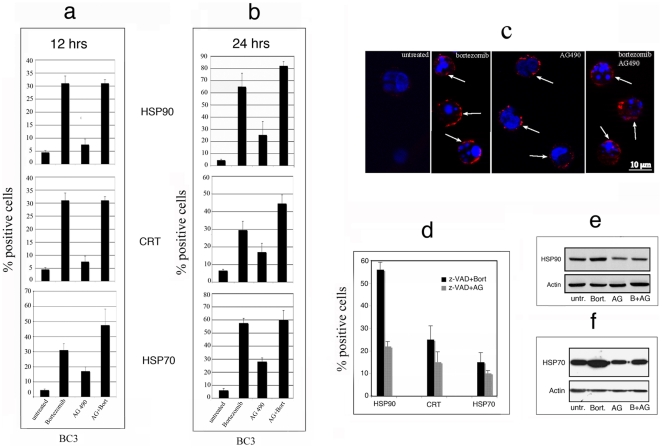
Bortezomib and AG 490 induce immunogenic BC3 cell death. Induction of HSP90, CRT and HSP70 traslocation on BC3 cell surface induced by Bortezomib, AG 490 or both after 12 hours (a) or 24 hours (b) treatments was evaluated by flow cytometric analysis. Mean of the percentage of positive cells plus SD of three independent experiments is indicated. Plasma membrane localization of CRT after Bortezomib, AG 490 or Bortezomib plus AG 490 treatments was also analyzed by immunofluorescence on BC3 fixed with 4% paraformaldehyde in PBS for 30 min and the arrows indicate the presence of CRT on the surface of the cells (c). One representative experiment out of three is shown. The effect of Z-VAD-fmk pre-treatment (100 µM) before exposure to Bortezomib and AG 490 on HSP90, CRT and HSP70 surface expression was evaluated by flow cytometric analysis. Mean of percentage of positive cells plus SD of three independent experiments is indicated (d). Western blot analysis showing the total HSP90 and 70 expression after 24 hours treatment of BC3 with Bortezomib, AG 490 or both (e–f). Results are representative of two independent experiments.

To analyze the immunizing properties of dead tumor cells, we evaluated the characteristics of the immunogenic cell death such as the surface expression of HSP70 and HSP90 and the relocation on the plasma membrane of the endoplasmic reticulum-resident CRT. The expression of these molecules was evaluated by FACS analysis after 12 and 24 hours of treatment with Bortezomib and AG 490, alone or in combination. PEL cells showed an higher surface expression of all molecules with Bortezomib alone or in combination with AG 490, compared to the single treatment with AG 490 after 12 hours (figure 3a). Moreover the percentage of positive cells increased after longer exposure (24 hours) for all treatments (figure 3b). Furthermore, intense CRT staining on the plasma membrane of BC3 cells was observed by immunofluorescence, after 24 hours with all the above treatments (figure 3c). We next evaluated whether caspase-dependent BC3 PEL cell apoptosis was involved in CRT, HSP90 and HSP70 surface translocation. Interestingly, Z-VAD-fmk pre-treatment, although reduced BC3 apoptosis (see above), did not impair the surface traslocation of CRT and HSP90, while reduced HSP70 surface expression (figure 3d). We next evaluated the total expression of HSP90 and HSP70 in BC3 cells treated with Bortezomib and AG 490 alone or in combination for 24 hours by immunoblotting. As shown in figure 3e, HSP90 expression was only slightly affected by drug treatments. On the contrary, Bortezomib alone increased the expression of HSP70 (figure 3f).

### Inhibition of DC maturation by blocking CD91, CRT, HSP90 and HSP70

**Figure 4:**
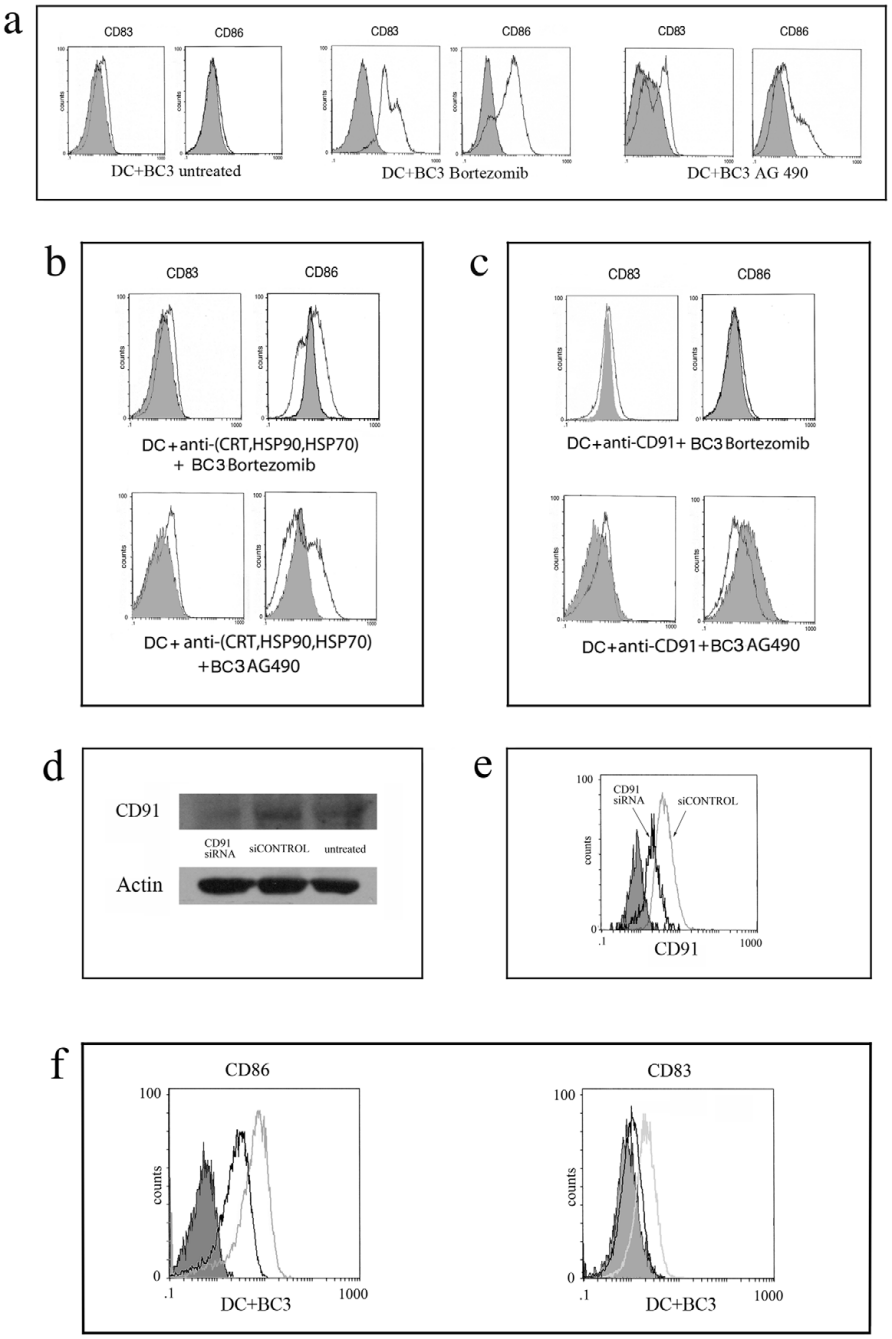
Inhibition of immunogenicity of Bortezomib and AG 490 killed BC3 PEL cells. DC activation obtained by coculture with BC3 cells untreated or killed by Bortezomib or AG 490 (a) or with killed BC3 coated with neutralizing antibody cocktail directed against HSP90, 70 and CRT before starting the coculture with iDC (b) or iDC coated with anti-CD91 mAb before starting the coculture with killed BC3 (c). Results are representative of three independent experiments. The staining with isotype control (filled histograms) is also shown. Reduction of CD91 expression by RNAi on DC was evaluated after 72 hours by Immunoblotting (d) and by FACS analysis (black empty hystogram in comparison with gray empty hystogram) (e). The effect of CD91 knockdown on the DC activation mediated by coculture with Bortezomib-killed BC3 cells (black empty histograms) in comparison with the control DC (gray empty histograms) (f). The staining with isotype antibody only (filled histograms) is also shown. Results are representative of three independent experiments.

We finally investigated the role of CRT and HSPs in the induction of DC maturation. To this end, BC3 treated with Bortezomib and AG 490 were coated with neutralizing antibodies directed against these molecules before coculturing with iDC. We found only a partial reduction in DC maturation, as assessed by CD83/CD86 staining, when a cocktail of antibodies for CRT, HSP90 and HSP70 was used (figure 4b) whereas the antibody directed against each single molecule did not have any inhibiting effect (data not shown). Although several molecules have been reported to act as receptors for HSPs or for HSP-peptide antigen complexes on the surface of DC, we observed a complete inhibition of DC maturation by using a neutralizing antibody directed against CD91, also known as LRP1, a common receptor for CRT, HSP90 and 70 (figure 4c). Altogether, these data indicate that Bortezomib and AG 490 induced traslocation on PEL BC3 surface of CRT, HSP90 and HSP70 and that DC maturation is mediated by the interaction of these molecules with CD91. To confirm the role of CD91 in the DC activation mediated by killed PEL we used RNAi to knockdown CD91 protein expression in human DC. To evaluate the silencing efficiency of CD91 we performed an immunoblot 72 hours after trasfection and as shown in figure 4d the CD91 expression was reduced respect to the control treated only with transfection reagent while the expression of control protein β-actin was unaffected. The flow cytometric analysis of CD91 siRNA displayed similar results (figure 4e). We next addressed the question of whether the knockdown of CD91 on DC had a negative effect on their activation after coculture with killed BC3 and as shown in figure 4f DC treated with CD91 siRNA displayed a reduction of the CD86 and CD83 markers. These results confirm the pivotal role of CD91 in the DC activation mediated by killed PEL.

## Discussion

Bortezomib has been shown to induce immunogenic cell death in solid tumors [22], in multiple myeloma due to the surface exposure of HSP90 [14] and to induce apoptosis in PEL [3]. Tyrphostin AG 490 has been also reported to induce apoptosis in PEL by inhibiting its constitutive STAT3 activation [5]. Here we asked if the Bortezomib- and AG 490-induced PEL cell death could be immunogenic. The immunogenicity of drugs-induced cell death was evaluated in terms of DC activation and maturation since DC have a pivotal role in the eradication of apoptotic cancer cells by mediating the cross-presentation of tumor antigens to the cytotoxic T cells, initiating a specific immune response [16]. For these reasons the DC activation by dying tumor cells is of fundamental importance for the outcome of cancer therapy [17]. The results shown in this paper demonstrate that Bortezomib and AG 490 induced apoptotic cell death in BC3 PEL with similar kinetic and that dying cells exposed CRT, HSP90 and HSP70 on the cell surface promoting uptake by DC and DC maturation. However, Bortezomib was more effective than AG 490 in inducing the expression of the above molecules and in inducing the DC uptake.

Apoptosis can have different impact on the immune system [6]. It is reported that apoptosis occurring during physiological tissue turnover does not result in the immune activation, however apoptosis can be immunogenic when induced by some cytotoxic agents [23]. Combining the killing effect of chemotherapies with the activation of immune response and in particular with DC activation is fundamental to improve the survival of cancer patients. Here we found that the drugs-induced BC3 cell death could be reduced by pre-treatment with pancaspase inhibitor Z-VAD-fmk. This is consistent with the finding that both drugs were able to induce apoptosis. Interestingly, the reduction of apoptosis by Z-VAD-fmk did not inhibit the maturation of DC, suggesting additional mechanisms other than caspase activation for triggering DC activation by the killed BC3 PEL cells. Essential for the immunogenicity of cell death seems to be the exposure of CRT, HSP90 and HSP70 on the surface of dying cells [9]. These molecules are usually located in the intracellular compartment where they regulate Ca2+ homeostasis (CRT) or have cytoprotective function (HSP90 and HSP70). When translocated on the cell surface they act as ‘damage-associated molecular patterns’ (DAMPs) and acquire immunostimulatory properties [6]. The membrane traslocation of CRT has been linked to ER stress and in particular to the PERK/eIF2α arm of the unfolded protein response (UPR) pathway. When exposed on the cell surface, CRT is a crucial determinant of the phagocytosis of tumor cells dying an apoptotic cell death and in the initiating a tumor-direct immune response [24], [25]. HSP90 and HSP70 are overexpressed in a ‘stressful environment’ such as tumor environment and under stress they can also translocate to the plasma membrane even if it is not clear through which mechanism [20], [26]. In agreement with these findings, we found that Bortezomib and AG 490 induced DC maturation through relocalization on PEL surface of CRT, HSP90 and HSP70.

These molecules, exposed on the plasma membrane of the stressed or dying cells, can interact with a number of receptors present on the DC surface that receive a direct activating signal or mediated by the HSP-peptide antigen complexes. Among them are Toll-like receptor 4 and 2, CD14, CD40, Lox-1 and CD91. The latter has an important role in the binding of CRT [27], HSPs [28] and has a foundament importance in the uptake and cross-presentation of chaperone-bound viral or tumor antigens [21]. Here we found that DC maturation obtained by cocultivating them with apopotic PEL BC3 cells was completely abolished by neutralizing CD91 receptor with a specific mAb antibody. The involvement of CD91 was further assessed by RNA interference showing strong reduction of DC maturation after CD91 knockdown. The importance of this molecule in the immune response has been underlied by the observation that CD91 represent an important route for stimulating a CD8^+^ T-cell response by MHC class I-restricted presentation [21]. Moreover, CD91 is the only molecule upregulated on the surface of DC in HIV-infected patients with no evidence of progressive disease for more than 10 years while remaining off treatment, termed “long-term nonprogressors” [29], [30]. Upregulation of CD91 in cancer patients may represent an additional strategy to improve DC activation and subsequent stimulation of a specific CD8^+^ T cell response towards tumor cells dying an immunogenic cell death. In conclusion, we show that Bortezomib and AG 490 induced BC3 PEL apoptosis and that apoptotic BC3 triggered DC maturation and phagocytosis. It is important to consider, though, that Bortezomib, if not extensively washed out before BC3 coculture with DC, had toxic effect on DC. This is consistent with previously reported studies [31]. On the contrary, AG 490 did not have any side effect on DC viability or function, suggesting a more risk-free use of AG 490 for systemic administation in cancer patients. Moreover, as STAT3 is hyperphosphorylated in DC in the tumor environment or exposed to the tumor supernatants [32], [33], leading to DC dysfunction, the use of AG 490 has been shown to be able to revert their dysfunction [34]. On the other hand, Bortezomib could be more indicated for target delivery to the tumor, such as by coupling it with tumor specific mAbs. Finally we observed that, although Bortezomib and AG 490 synergized in inducing BC3 PEL cell death, their combination did not result in a better activation of DC and this observation warrants caution in cancer therapies combining both molecules, already in clinical trials for some tumors [35].
